# Dual-specificity tyrosine-regulated kinase 4 modulates the STAT3-FOS signaling axis to inhibit hepatitis B virus replication *via* autophagy

**DOI:** 10.7150/ijbs.105447

**Published:** 2025-03-10

**Authors:** Jiaqi Xu, Xianhuang Zeng, Junsong Huang, Shuangshuang Ma, Kun Li, Siqi Yang, Wajeeha Naz, Tanzeel Yousaf, Sen Yuan, Yang Liu, Jing Zhang, Chaozhi Liu, Chenyi Liu, Zixu Zhai, Mingxiong Guo, Guihong Sun

**Affiliations:** 1 TaiKang Medical School (School of Basic Medical Sciences), Wuhan University, Wuhan 430071, Hubei, P.R. China.; 2 Hubei Key Laboratory of Cell Homeostasis, College of Life Sciences, Wuhan University, Wuhan 430072, Hubei, P.R. China.; 3 School of Ecology and Environment, Tibet University, Lhasa 850000, Xizang, P.R. China.; 4 Hubei Provincial Key Laboratory of Allergy and Immunology, Wuhan 430071, Hubei, P.R. China.

**Keywords:** HBV replication, DYRK4, STAT3, FOS, autophagy

## Abstract

Chronic hepatitis B virus (HBV) infection is a major global cause of hepatocellular carcinoma (HCC). Despite available antiviral strategies, the therapeutic eradication of HBV from infected cells remains challenging. Recent studies have highlighted the role of dual-specificity tyrosine-regulated kinases (DYRKs) in innate immunity against viruses and HCC; however, the antiviral function of DYRK4 against HBV infection remains unknown. Here, we report that DYRK4 efficiently inhibited HBV replication both *in vitro* and *in vivo*. Mechanistically, we demonstrate a direct interaction between TAB1 (TGF-beta activated kinase 1 [MAP3K7] binding protein 1) and the kinase domain of DYRK4, which may inhibit HBV replication. Importantly, we found that the kinase activity of DYRK4 plays a key role in inhibiting HBV replication *via* its K133 site. Further, we revealed that DYRK4-induced STAT3 ubiquitination degradation results in decreased STAT3 translocation into the nucleus. Subsequently, this reduction in STAT3 downregulates FOS expression to decrease autophagy-inducible factor BECN1 (Beclin1) and LC3 I/II expression, which inhibited HBV replication *via* autophagy. Overall, these findings identify a novel antiviral function of DYRK4 against HBV replication*.* The ability of the DYRK4-K133 kinase activity to downregulate autophagy *via* STAT3-FOS axis presents a potential therapeutic target for hepatitis B.

## Introduction

Chronic hepatitis B virus (HBV) infection is the main cause of hepatocellular carcinoma (HCC), affecting approximately 296 million individuals worldwide and resulting in 1.5 million new infections annually 1. Although current therapies can hinder the disease, they cannot eliminate HBV completely due to the integration of the HBV covalently closed circular DNA (cccDNA) into the host genome, which serves as the template to re-initiate the virus replication after prolonged viral suppression 2. Therefore, further comprehensive research is necessary to identify novel host restriction factors involved in HBV disease progression in order to attain a functional cure for HBV, which the World Health Organization aims to achieve by 2030 3.

HBV, an enveloped DNA virus belonging to the Hepadnaviridae family, possesses two unequal 3.2 kb DNA strands that form an incomplete loop known as relaxed circular DNA (rcDNA). Upon entry into the host hepatocytes, the virus undergoes uncoating, leading to the complete closure of the rcDNA and formation of covalently closed circular DNA (cccDNA), which is capable of integrating into the genomic DNA of the host hepatocyte 2, 4. The persistence of cccDNA within infected hepatocyte nuclei is responsible for chronic HBV replication 2. Subsequently, cccDNA serves as a template for the transcription and production of viral RNAs of differing lengths: 3.5-kb preC RNA, 3.5-kb pgRNA, 2.4-kb preS1 RNA, 2.1-kb preS2 RNA, and 0.7-kb HBx RNA 5. Translation of these RNAs results in the synthesis of viral proteins, including the hepatitis B core antigen (or nucleocapsid protein), soluble HBeAg secretion protein, polymerase protein, envelope protein referred to as HBsAg, and hepatitis B X protein 5.

Dual-specificity tyrosine-regulated kinase 4 (DYRK4) is a member of the DYRK family, which comprised of serine/threonine (Ser/Thr) protein kinases. This family shares a highly conserved amino acid sequence in the catalytic domain, including a Tyr-X-Tyr motif in the activation loop 6. There are limited number of studies regarding DYRK4 in cell development, nerves, tumors, and immunity as compared to other DYRK family members, such as DYRK2 and DYRK3, which are associated with HCC development 6-8. DYRK2 has been shown to be involved in innate immune regulation and viral infection 9, and has a potential role in protein phosphorylation and degradation 9, 10. According to its classification alongside DYRK2 and DYRK3, it is possible that DYRK4 also participates in physiological activities such as innate immunity, antiviral response, or liver cancer. We have previously reported that DYRK4 can upregulate antiviral innate immunity 11, and a previous study already reported increased expression of DYRK4 in HCC 12. Interestingly, chronic HBV replication is the primary risk factor for HCC development. Thus, DYRK4 upregulation in HCC in prior studies inspired us to investigate the relationship between DYRK4 and HBV replication.

Our findings provide insights into the mechanisms underlying anti-HBV effects of DYRK4. We provided evidence of a direct interaction between TAB1 and the kinase domain of DYRK4, which may inhibit HBV replication. Furthermore, through modulation of the STAT3-FOS axis, we show that DYRK4 downregulates autophagy and ultimately inhibits HBV replication. We found that the K133 site of DYRK4 plays a key role in inhibiting HBV replication. In summary, our findings uncover a previously unknown antiviral role of DYRK4 against HBV *via* regulation of autophagy.

## Materials and Methods

### Cell cultures

The Huh7, HepG2, HepG2-NTCP, and HCCLM3 human hepatoma cells were cultured in DMEM supplemented with 10% fetal bovine serum. HepG2.2.15 cells were treated and cultured in DMEM containing 400 μg/mL G418. HepG2-NTCP cells were maintained in the same medium but with a pre-coated layer of 50 μg/mL rat tail collagen type 1 (Corning ®) on the bottom surface. All cell lines were incubated at 37 ℃ in humidified CO_2_ (5%) incubators.

### Northern and Southern blot analysis

The cells were co-transfected with plasmids for at least 48 h. The RNA was then subjected to electrophoresis on a 1% formaldehyde agarose gel and subsequently transferred onto a nylon membrane for hybridization with the HBV-DIG probe. Additionally, intracellular HBV core particle-associated DNA was separated on a 1% agarose gel and transferred onto a nylon membrane for hybridization using the HBV-DIG probe. Detection of the blot was performed using the DIG Starter Kit (Roche Diagnostics, Indianapolis, IN), which targeted nucleotides 1072**-**2171 of the HBV genome for Northern blot analysis and nucleotides 157**-**1068 of the HBV genome for Southern blot analysis 13.

### Detection of cccDNA

Methods for the extraction of total DNA from cells and detection of cccDNA were as previously described 14, 15. Using a Genomic DNA Kit from Tiangen Technology (Beijing) Co., Ltd., the DNA concentration was measured using NanoDrop 2000 (Thermo Scientific), and then the total amount of DNA was normalized. Then real-time quantitative polymerase chain reaction (qRT-PCR) was performed using cccDNA-specific primers 14, and the results were compared after normalizing the cycle thresholds.

### RNA extraction and qRT-PCR

Total RNA was extracted from cells and tissues using Trizol (Invitrogen) and chloroform, followed by reverse transcription to cDNA using the PrimeScript RT Reagent Kit (TAKARA). qRT-PCR analysis was performed with gene primers obtained from Wuhan Tianyi Huayu Gene Technology, and the SYBR Green dye was provided by Yeasen, China. The results were acquired using the CFX96TM Real-time System software version 3.1 (Bio-Rad). All primer sequences are listed in the supplemental table **(Table S1)**.

### Enzyme-linked immunosorbent assay (ELISA)

Huh7 cells and HepG2.2.15 cells were transfected with the specific plasmids. The levels of HBV surface antigen (HBsAg) and HBV e antigen (HBeAg) in the supernatant were quantified using an enzyme-linked immunosorbent assay (ELISA) kit (Wantai Biology, China), and measured using a microplate reader (Biotek, Synergy H1).

### Observation of autophagy by confocal microscope

HepG2.2.15 cells were seeded into 12-well plates and transfected for 48 h. The cells were then transferred to a confocal dish to maintain a cell density of approximately 20% for 24 h. The cells were fixed with 4% paraformaldehyde for 20 min, and then washed with 1 × PBS to remove the fixative solution. The cells were treated with 0.2% Triton X-100 for 15 min to enhance permeability. DAPI (4',6'-diamino-2-phenylindole) was added for approximately 5 min to stain the nuclei, followed by another wash containing only 0.02% Triton X-100 for 10 min. Finally, the samples were placed in PBS, stored in the dark, and observed under a confocal microscope (Leica TCS SP8).

### Hydrodynamics-based transfection in mice

A previously described HBV infection mouse model was utilized in this study 13. male BALB/c mice (10**-**11 weeks) and male C57BL/6 mice (11**-**12 weeks) were purchased from the Hubei Provincial Center for Disease Control and Prevention (Wuhan, China). Each plasmid (10 μg) was premixed with 2 mL of PBS and injected in mice via their tail vein within 6**-**8 seconds. All mice were housed in a pathogen-free animal colony, and all animal experiments in this study were conducted following animal welfare and ethical regulations as reviewed by the IACUC of Wuhan University (No. WDSKY0201409-2).

### Glutathione-S-transferase (GST) pull-down assay

Protein expression was induced in Transetta (DE3) cells using 0.2 mM isopropyl β-D-thiogalactoside (IPTG) at 16 °C for 20 h. The bacteria were lysed, and the target protein was purified using a GST- or His-tag system. Then the target proteins were co-incubated with GST beads at 4 °C for 6 h. Then the GST beads were washed with PBS and resolved by SDS-PAGE.

### Extraction of intracellular HBV core particle-associated DNA

This method was described previously 13. Cells were lysed with 1% NP-40 (Beyotime, P0013F) on ice, followed by adding DNase I and RNase A to the supernatant and incubating in a water bath at 37°C. Proteinase K and 20% SDS were then added, and the cells were incubated at 55°C for 8 h. The resulting mixture was then extracted using a 1:1 ratio of phenol and chloroform, followed by centrifugation to obtain the supernatant. Anhydrous ethanol and NaAc (3 M, pH = 5.2) were added to the supernatant and incubated at 25 °C for 5 min. The sediment was retained and washed with 75% ethanol before being dissolved in distilled water.

### Co-immunoprecipitation

Cells were carefully collected in lysis buffer (Beyotime, P0013 Cell Lysis Buffer) supplemented with a protease inhibitor cocktail. The samples were centrifuged and the supernatants were collected. An aliquot of the samples was used as the input sample. Washed Flag-beads were added to the supernatants and incubated at 4 °C incubation for 6 h. After that, the supernatant was discarded and 1 mL of washing buffer was added. The samples were then washed for five times. Then 2 × loading buffer was added to the samples, and the samples were boiled at 100 °C for 5 min.

### Statistical analysis and drawing

All experiments were performed at least three times. Statistical significance was determined by using an unpaired Student's t-test between two groups (* *P* < 0.05, ** *P* < 0.01, *** *P* < 0.001). All data analyses were performed by using the statistical analysis software ImageJ and GraphPad Prism (GraphPad Software, San Diego, CA). Data images, drawings, and layouts were completed using Adobe Photoshop 2024 and Adobe Illustrator 2024. The graphical abstract was completed by using Figdraw (https://www.figdraw.com).

## Results

### DYRK4 suppresses HBV replication

**Figure 1:**
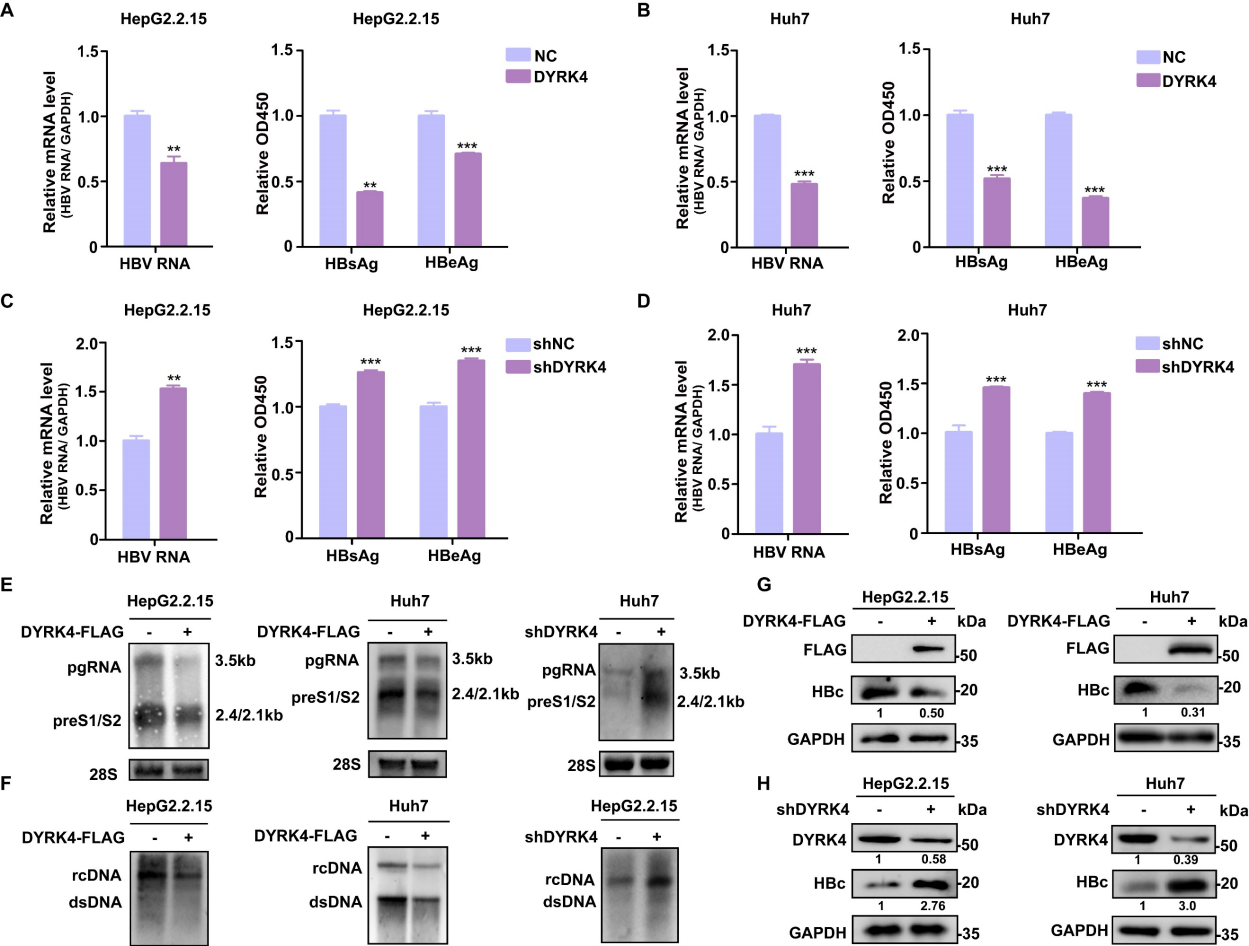
** DYRK4 suppresses HBV replication *in vitro*.** (A-D) Overexpression or knockdown of DYRK4 in HepG2.2.15 cells and Huh7 cells were conducted to detect the HBV nuclear acid and HBsAg/HBeAg levels. (A) HepG2.2.15 cells were transfected with the DYRK4-FLAG plasmid, and (B) Huh7 cells were transfected with the DYRK4-FLAG plasmid and the pHBV1.3 plasmid for 48 h. (C) HepG2.2.15 cells were transfected with the shDYRK4 plasmid, and (D) Huh7 cells were transfected with the shDYRK4 plasmid with pHBV1.3 for 48 h. Total RNA was extracted from the cells and HBV mRNA was subjected to qRT-PCR analysis. GAPDH served as the loading control. The cell supernatant was harvested from the culture and used to evaluate HBsAg and HBeAg levels through ELISA at OD450, ** *P* < 0.01, *** *P* < 0.001. (E) HBV RNA was extracted from these cells and analyzed by Northern blot with an HBV-DIG probe. (F) Detection of HBV core-associated DNA (rcDNA, dsDNA) was conducted by Southern blot analysis. (G-H) Western blot analysis was performed to detect HBc and DYRK4 protein levels in HepG2.2.15 cells and Huh7 cells overexpressing DYRK4 (G) or with DYRK4 knockdown (H).

To investigate the potential influence of DYRK4 on HBV replication, we utilized overexpression and knockdown strategies in HBV-stably-transfected HepG2.2.15 cells and in HBV-transfected Huh7 cells **(Fig. S1).** The results showed that overexpression of DYRK4 significantly suppressed HBV replication by inhibiting the total RNA, as well as the secreted HBsAg, and HBeAg levels **(Fig. 1A-1B)**. Conversely, knockdown of endogenous DYRK4 using short hairpin RNA (shRNA) resulted in enhanced HBV total RNA and increased secretion of HBsAg and HBeAg **(Fig. 1C-1D)**. Similar results were obtained in Northern blot and Southern blot, overexpression of DYRK4 inhibited HBV pre-genomic RNA (pgRNA), preS1/S2 RNA levels** (Fig. 1E)**, and HBV core particle-associated DNA levels **(Fig. 1F)**. Furthermore, HBc protein expression was significantly reduced by DYRK4** (Fig. 1G)**, while knockdown of DYRK4 yielded the opposite results** (Fig. 1H)**. Together, these findings indicated that DYRK4 substantially inhibits HBV replication.

### DYRK4 suppresses HBV replication by reducing autophagy

Previous research has shown that autophagy is beneficial in the replication of HBV 13. We also verified the significance of autophagy in HBV infection in HepG2-NTCP cells, by employing the autophagy inhibitor, 3-methyladenine (3-MA) 16. Western blot analysis demonstrated that BECN1 was reduced by 3-MA, thus the inhibitor suppressed autophagy **(Fig. S2A)**. We further detected the HBV DNA present in the genomic DNA of cells' DNA by qRT-PCR. We found that the HBV DNA levels were reduced by the autophagy inhibitor **(Fig. S2B)**. Moreover, ELISA results showed that the HBsAg/HBeAg levels in cell supernatants were decreased **(Fig. S2C)**.

**Figure 2:**
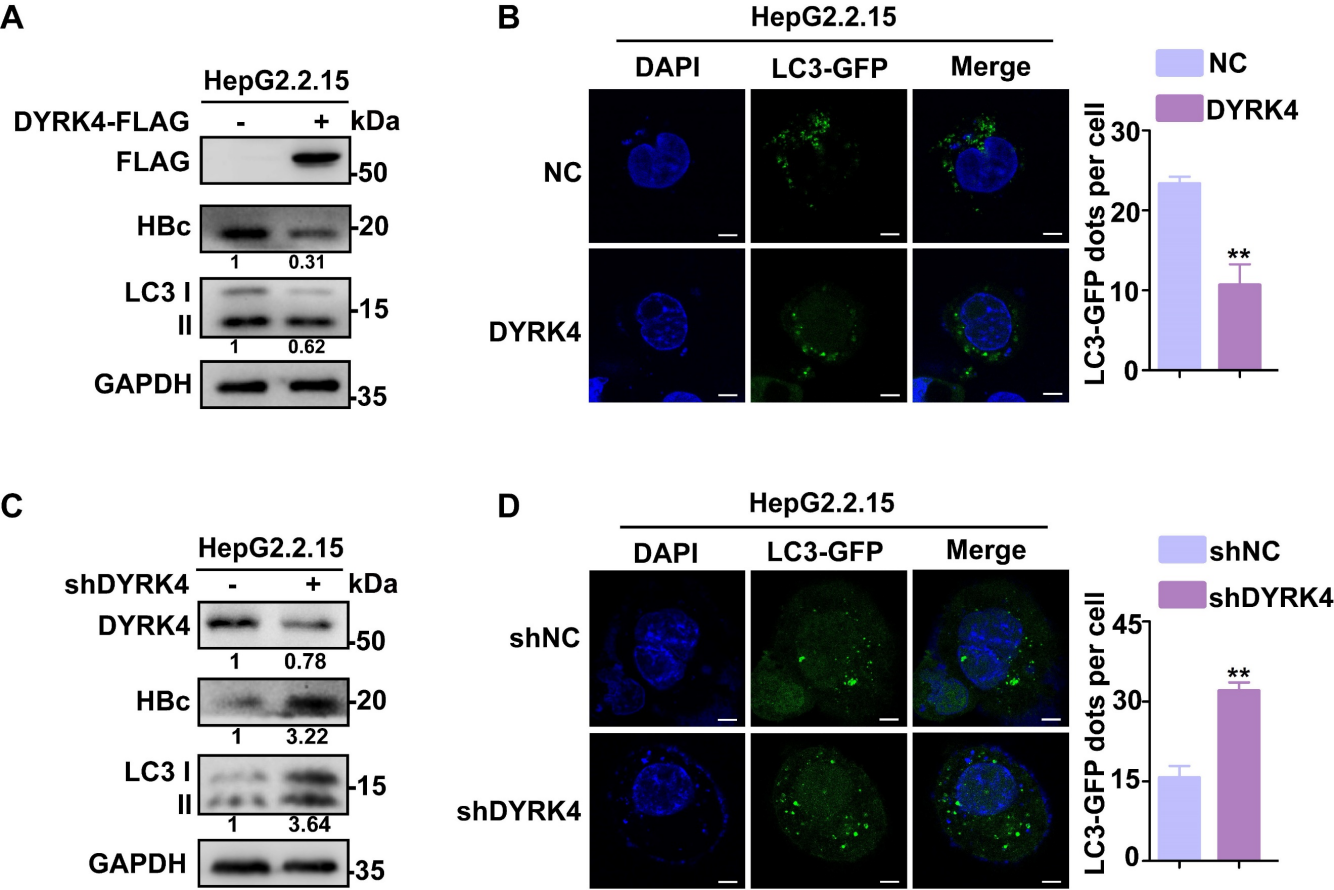
** DYRK4 suppresses HBV replication by inhibiting autophagy.** (A-B) Overexpression of DYRK4 was conducted in HepG2.2.15 cells. The protein levels of LC3 I/II and HBc were detected by Western blot. Subsequently, LC3-GFP fluorescent autophagosomes were observed and quantified *via* confocal microscopy. (C-D) Knockdown of DYRK4 was conducted in HepG2.2.15 cells by shRNA. The protein levels of LC3 I/II and HBc were detected by Western blot. LC3-GFP dots were observed and quantified using confocal microscopy. Scale bar: 5 μm. The number of LC3-GFP dots in three replicates were analyzed by ImageJ software, ** *P* < 0.01.

To elucidate if DYRK4 suppresses HBV replication by reducing autophagy, we detected the changes in the expression levels of total microtubule-associated protein 1 light chain 3 I/II (LC3 I/II) after DYRK4 overexpression or knockdown. Total LC3 I/II expression was obviously decreased by DYRK4, along with HBc **(Fig. 2A)**, and confocal microscopy further confirmed these findings by demonstrating a significantly lower number of LC3-GFP fluorescent autophagosomes in DYRK4 overexpressing HepG2.2.15 cells **(Fig. 2B)**. Conversely, DYRK4 knockdown significantly increased both the total LC3 I/II expression **(Fig. 2C)** and the number of LC3-GFP-labeled autophagosomes **(Fig. 2D)**.

**Figure 3:**
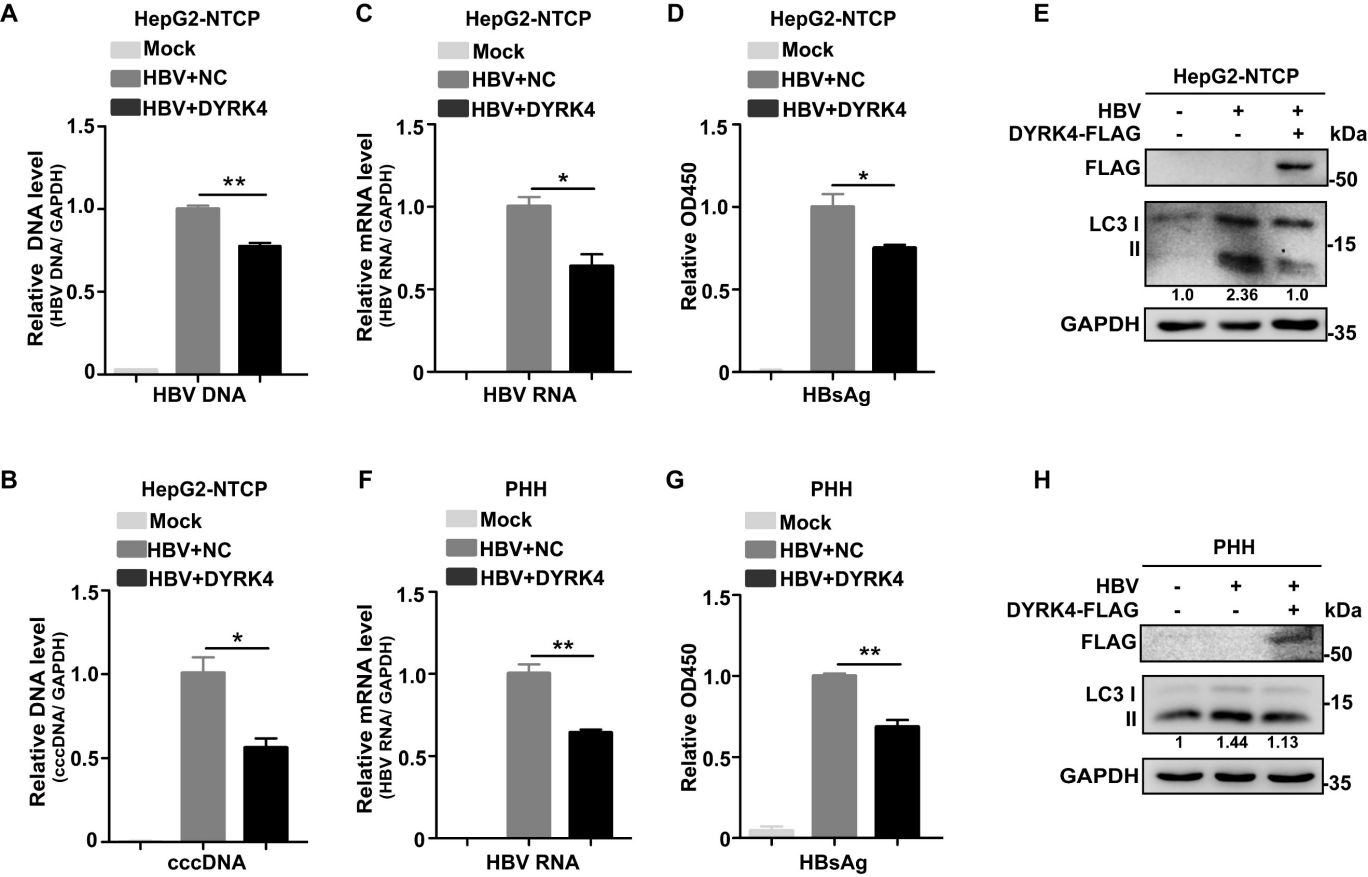
** DYRK4 suppresses HBV replication after infection.** (A-B) HepG2-NTCP cells were infected with HBV (MOI = 200) for five days and then transfected with the DYRK4-FLAG plasmid for two days. The total HBV genomic DNA was extracted and assessed by qRT-PCR using HBV DNA and cccDNA-specific primers. A GAPDH DNA primer served as a loading control. (C-E) HepG2-NTCP cells were infected with HBV (MOI = 200) for five days and transfected with the DYRK4-FLAG plasmid for two days. (C) The total HBV mRNA level was estimated by qRT-PCR, and GAPDH served as the loading control. (D) The HBs antigen was detected by ELISA. (E) The protein level of LC3 I/II were detected by Western blot analysis. (F-H) PHH cells were infected with HBV (MOI = 200) for five days and transfected with the DYRK4-FLAG plasmid for two days. (F) The HBV mRNA level was estimated by qRT-PCR, and GAPDH served as the loading control. (G) The HBs antigen was detected by ELISA. (H) The protein level of LC3 I/II was analyzed by Western blot. * *P* < 0.05, ** *P* < 0.01.

Additionally, to confirm that DYRK4 inhibits HBV replication, we performed HBV infection experiments. After successful HBV infection in HepG2-NTCP cells, DYRK4 plasmids were transfected into the cells and HBV replication was assessed. The results demonstrated that after HBV infection, overexpression of DYRK4 downregulated the relative HBV genomic DNA **(Fig. 3A)** and cccDNA levels **(Fig. 3B)**, along with the total RNA **(Fig. 3C)** and secreted HBsAg levels **(Fig. 3D).** Moreover, overexpression of DYRK4 also reduced the expression of LC3 I/II, which is a marker of autophagy **(Fig. 3E)**. In HBV infected primary human hepatocytes (PHHs), we found that the anti-HBV function of DYRK4 was consistent with the findings in HBV-infected HepG2-NTCP cells **(Fig. 3F-3H)**. These results indicated that DYRK4 inhibits HBV replication *via* autophagy.

### DYRK4 directly interacts with TAB1

**Figure 4:**
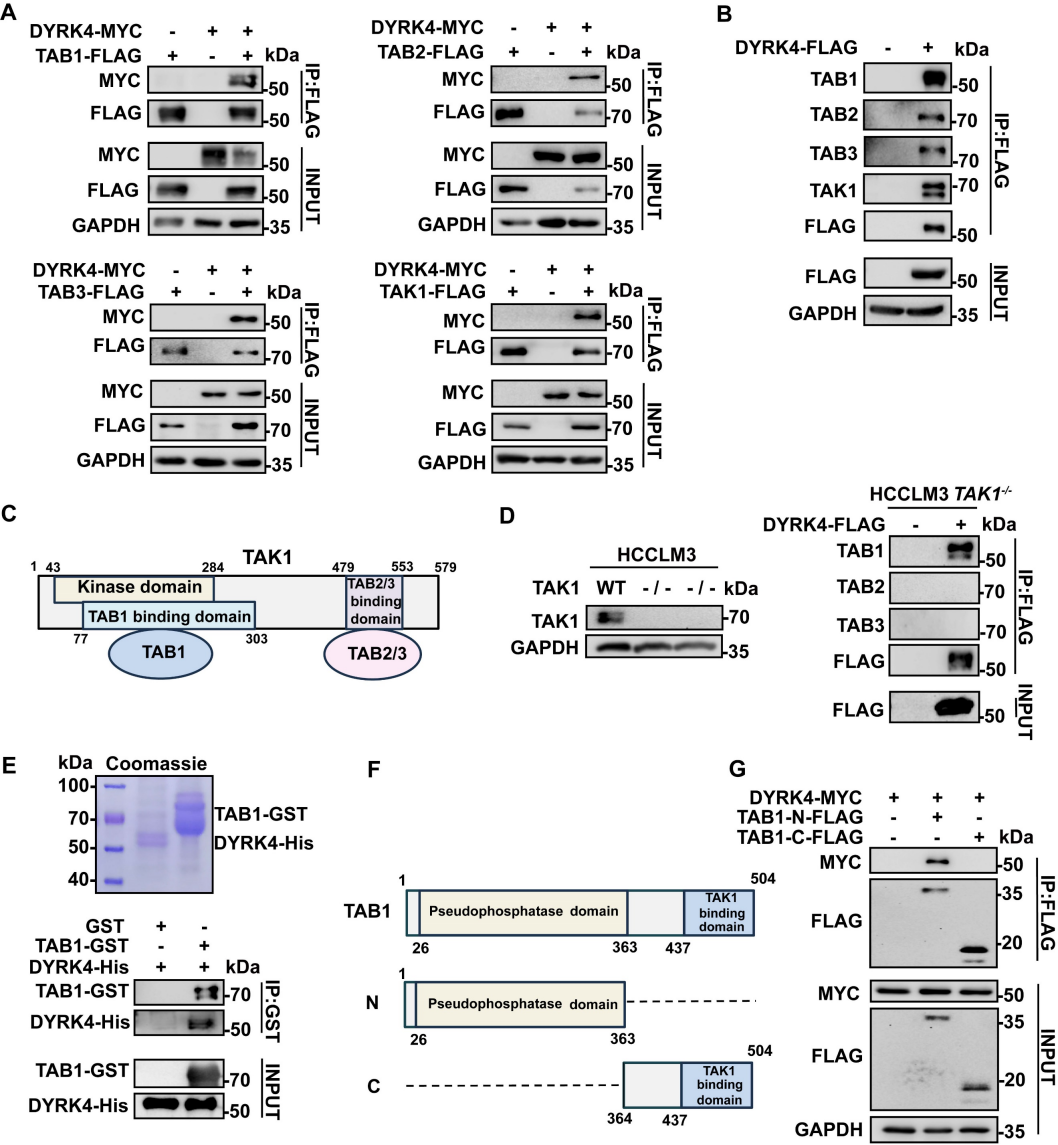
** DYRK4 directly interacts with TAB1.** (A) Co-IP experiments were conducted in Huh7 cells with the pHBV1.3 plasmid, demonstrating the interaction between overexpressed TAK1-TABs and DYRK4. (B) DYRK4 was overexpressed in Huh7 cells with the pHBV1.3 plasmid for 48 h. The cells were then subjected to co-IP analysis with endogenous TAK1-TABs. (C) A structural diagram of the TAK1-TABs complex. (D) Co-IP experiments revealed the interaction between DYRK4 and TABs in a *TAK1*^-/-^ HCCLM3 cell line transfected with the pHBV1.3 plasmid. (E) GST-pull-down assays were performed, followed by SDS-PAGE and Western blot analysis. Purified DYRK4-His and TAB1-GST proteins were visualized using Coomassie brilliant blue staining on SDS-PAGE gel, and their interaction was detected through Western blot. (F) A structural diagram of TAB1, along with information about its different segments. The N-domain (1-363 aa) includes a pseudo-phosphorylation domain, and the C-domain (364-504 aa), is known as the TAK1 binding domain. (G) Co-IP between the TAB1 N-domain, C-domain and DYRK4 in Huh7 cells transfected with the pHBV1.3 plasmid.

To elucidate the mechanism underlying the inhibition of HBV replication by DYRK4, we analyzed DYRK4 mass spectrometry results from our previous report 11. The mass spectrometry result indicated that DYRK4 interacted with the TGF-beta-activated kinase 1/MAP3K7 binding proteins (TAB1/2/3) complex 11. The references illustrated that TAB1/2/3 formed the complex with TAK1 17, so it is reasonable to hypothesize that DYRK4 might interact with the various components of the TAB1/2/3-TAK1 complex. To validate the mass spectrometry findings and our hypothesis, the co-IP assays were performed, which confirmed the interaction between DYRK4, TAB1/2/3, and TAK1 **(Fig. 4A)**. Furthermore, overexpression of DYRK4 also interacted with endogenous TABs and TAK1 **(Fig. 4B)**. Based on the high score in the Sequest HT of TAB1 as compared to TAB2/3 in the mass spectrometry analysis and the structural relationship between TAK1 and TABs **(Fig. 4C)** 17, we theorized if TAB1 acts as a “bridge” facilitating interactions between DYRK4 and TAB2/3 *via* TAK1. To address this speculation, we constructed *TAK1*-knockout HCCLM3 cells using CRISPR-Cas9 technology. Notably, TAB1 was found to still interact with DYRK4 in* TAK1*^-/-^ HCCLM3 cells **(Fig. 4D)**. Importantly, the overexpression of DYRK4 remained inhibitory to HBV replication in *TAK1*-knockout cell line **(Fig. S3A)**. Conversely, the downregulation of DYRK4 exerted an opposite effect **(Fig. S3B)**, suggesting that the inhibition of DYRK4-induced HBV replication may not rely solely on the TAK1 pathway. Further, to check whether DYRK4 directly interacts with TAB1 *in vitro*, a pull-down assay was performed using purified His-tagged DYRK4 protein and GST-tagged TAB1 protein **(Fig. 4E, upper panel)**. The result demonstrated that TAB1-GST successfully pulled down DYRK4-His, as compared to the control GST **(Fig. 4E, lower panel)**. Based on the structure of TAB1, the C-terminal TAK1 binding domain and the N-terminal pseudophosphastase domain 18 **(Fig. 4F)**, we constructed a plasmid containing either the N- or C- terminal domain. Co-IP experiment revealed that DYRK4 specifically interacted with the N-terminal pseudophosphastase domain of TAB1 **(Fig. 4G)**. These results indicated that DYRK4 directly interacted with TAB1 and inhibited HBV replication independent of TAK1 and TAB2/3.

### TAB1 influences DYRK4 to inhibit HBV replication

**Figure 5:**
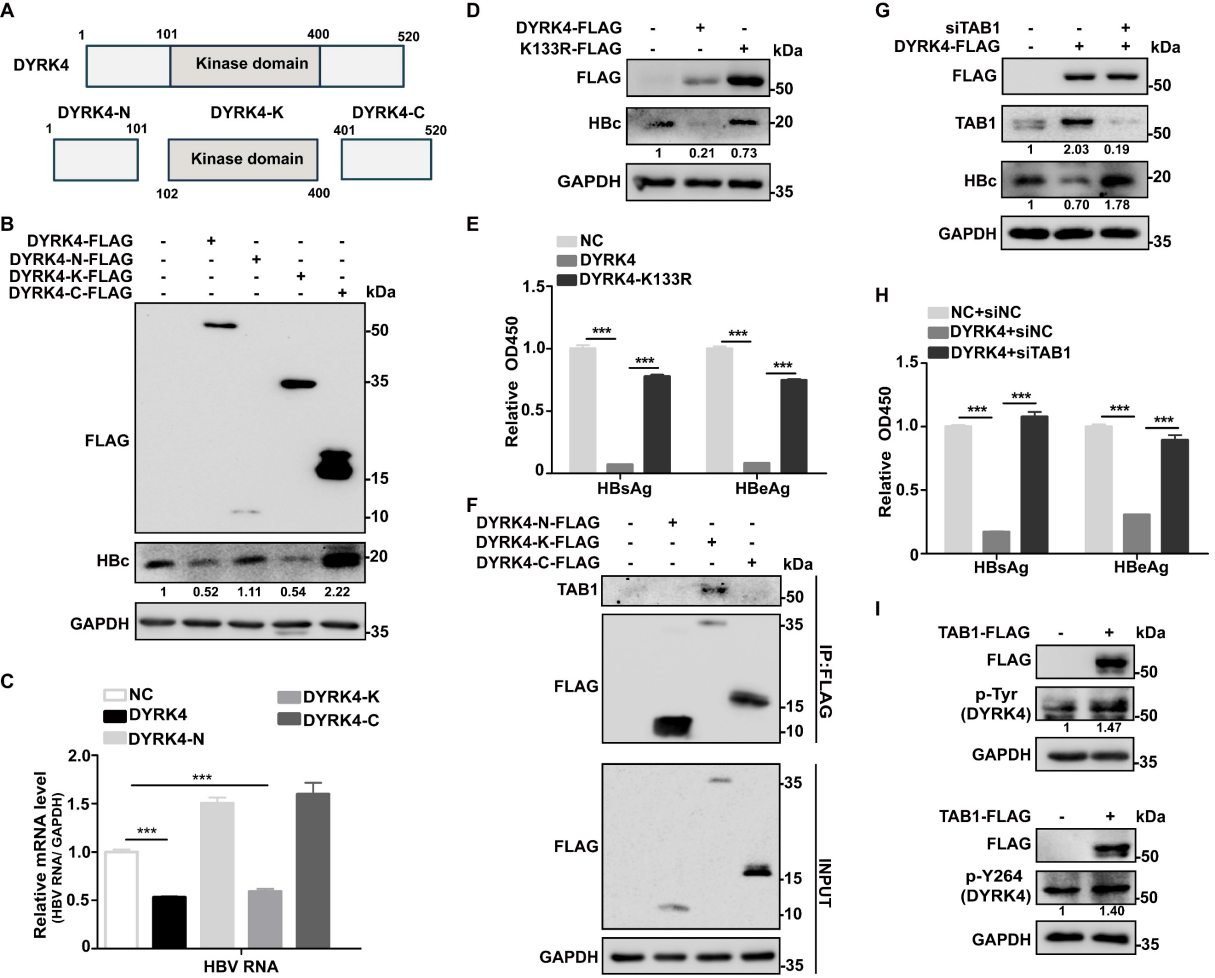
** TAB1 influences DYRK4 to inhibit HBV replication.** (A) The structure of DYRK4 is divided into three domains: the N-domain (1-101 aa), which includes the NAPA-domain (N-terminal autophosphorylation accessory domain), DH-BOX (DYRK homology box), and NLS (nuclear localization sequence); the K-domain (102-400 aa), which contains the entire kinase activity domain; and the C-domain (401-520 aa), whose function is still unclear. (B-C) Huh7 cells were co-transfected with DYRK4 truncation plasmids and the pHBV1.3 plasmid for 48 h, and Western blot analysis was performed to detect the expression of DYRK4 truncations and HBc protein. qRT-PCR was used to estimate the level of HBV RNA. GAPDH was served as the loading control. (D-E) Mutant DYRK4-K133R amino acid plasmid was constructed. After expressing this mutation in Huh7 cells, Western blot (D) and ELISA (E) were performed to analyze HBc, HBsAg, and HBeAg expression levels. (F) The DYRK4 truncation plasmids and the pHBV1.3 plasmid were co-transfected in Huh7 cells for 48 h. Then the cells were prepared for co-IP analysis of DYRK4 truncations with TAB1 using FLAG-beads enrichment. (G-H) Co-transfection of DYRK4-FLAG plasmid with siTAB1 in Huh7 cells along with the pHBV1.3 plasmid enabled detection of HBsAg, HBeAg and HBc expression levels by ELISA (H) and Western blot (G). (I) Phosphorylation at tyrosine and Y264 residue on DYRK4 were specifically examined by Western blot when TAB1 was overexpressed in pHBV1.3-transfected Huh7 cells. *** *P* < 0.001.

Since DYRK4 is a kinase protein, we were interested in determining if DYRK4 inhibited HBV replication *via* its kinase activity. To delineate this, we constructed three truncated domains - the N, Kinase (K), and C domains **(Fig. 5A)** - based on previous studies 19. We co-transfected these truncated domains along with the pHBV1.3 plasmids into Huh7 cells and found that the wild-type and kinase domains of DYRK4 effectively suppressed both HBc and HBV RNA, while the N and C terminal domains did not exhibit any inhibition **(Fig. 5B-5C)**. These results strongly indicated that the kinase activity of DYRK4 was pivotal in the suppression of HBV. To further address the importance of DYRK4 kinase activity in HBV replication, we constructed a catalytically dead K133R mutant of DYRK4 20, 21. The mutant variant displayed a significant reduction in kinase activity and was unable to inhibit HBV replication as compared to wild-type DYRK4 **(Fig. 5D-5E)**. Similar results were verified in regard to HBV DNA in the supernatant of HepG2.2.15 cells **(Fig. S4)**. These findings confirmed that the kinase activity of DYRK4 plays a key role in suppressing HBV.

Previous studies have revealed that TAB1 interacts with and activates TAK1 and p38α by mediating their autophosphorylation 22, 23. Based on these studies, we proposed that the interaction between TAB1 and DYRK4 may enhance the kinase activity of DYRK4 and inhibit HBV replication. A co-IP assay showed that TAB1 interacted only with the kinase domain and not with the N- or C- terminal domains of DYRK4 **(Fig. 5F)**. We overexpressed DYRK4 and knocked down TAB1 by siRNA in pHBV1.3-transfected Huh7 cells and showed that TAB1 knockdown markedly restored the inhibitory effect of DYRK4 on HBc expression and HBs/HBe secretion **(Fig. 5G-5H)**. Additionally, we observed by confocal microscopy in HepG2.2.15 cells, that DYRK4 overexpression inhibited the number of autophagosomes, and TAB1 knockdown recovered the number of autophagosomes **(Fig. S5)**, which was consistent with HBV replication. Further, TAB1 enhanced the total phosphorylation of DYRK4 including at the Y264 site (which is an autophosphorylation site of DYRK4) **(Fig. 5I)**. These findings substantiated that TAB1 may play an important role in the kinase activity of DYRK4.

### DYRK4 inhibits HBV replication by suppressing the STAT3-FOS signaling axis

**Figure 6:**
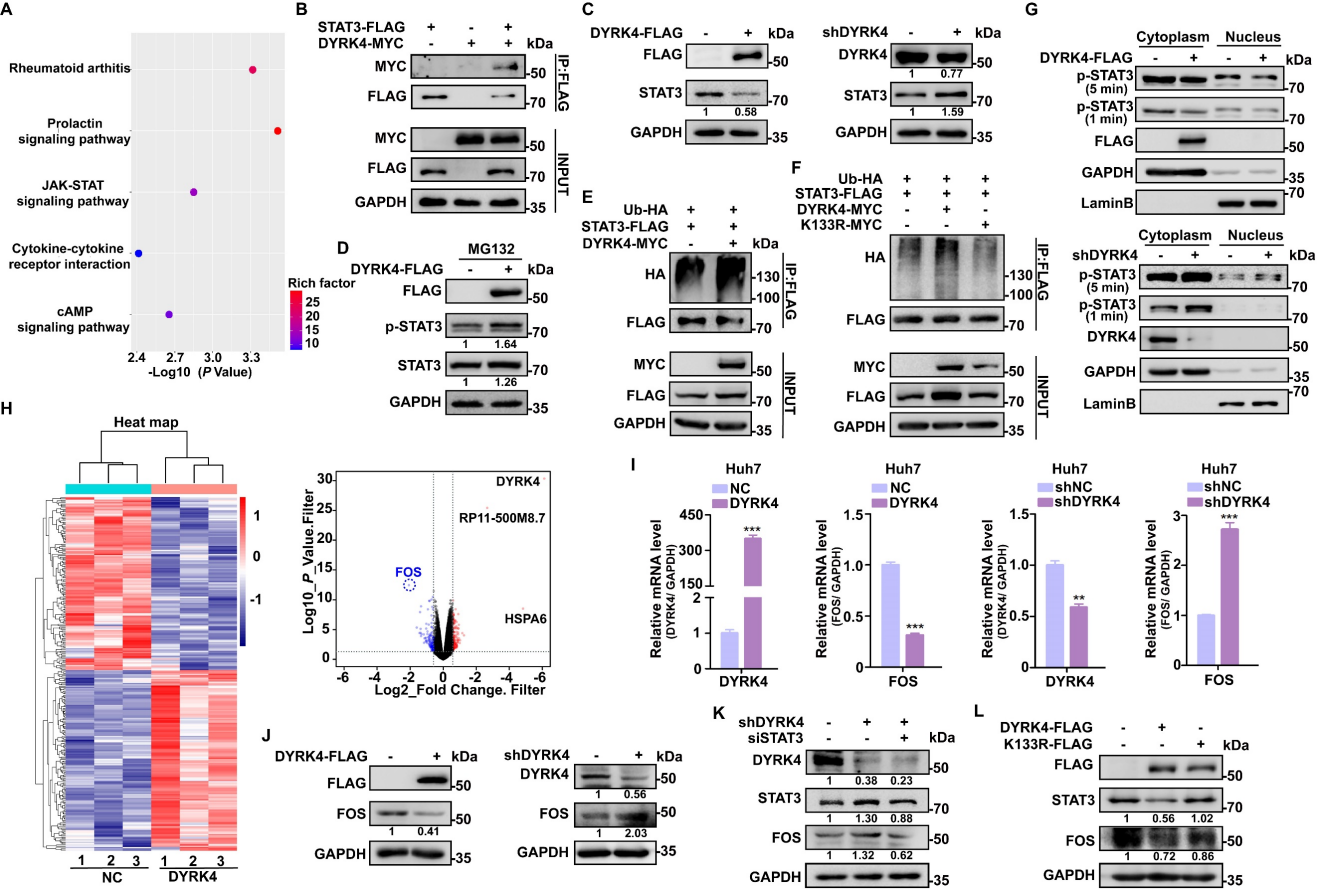
** DYRK4 inhibits HBV replication by suppressing the STAT3-FOS signaling axis.** (A) The Kyoto Encyclopedia of Genes and Genomes (KEGG) signaling pathway. (B) The interaction between DYRK4 and STAT3 in Huh7 cells was demonstrated by a co-IP assay. (C) Huh7 cells were co-transfected with the DYRK4-FLAG or shDYRK4 plasmids along with pHBV1.3 for 48 h, followed by Western blot analysis to detect the expression of STAT3. (D) Western blot analysis detected the levels of STAT3 and phospho-STAT3 proteins under MG132 (10 μM) treatment while DYRK4 was overexpressed. (E-F) Western blot analysis revealed the ubiquitin-mediated degradation of STAT3 in the presence of DYRK4 and DYRK4-K133R after culturing plasmids for 48 h in Huh7 cells with the pHBV1.3 plasmid. (G) After co-transfection of DYRK4-FLAG or shDYRK4 with the pHBV1.3 plasmid in Huh7 cells, the nuclear translocation of phosphorylated STAT3 was detected by Western blot following nuclear and cytoplasmic fractionation. (H) A heatmap and volcano plot were generated from the RNA-sequencing data obtained from HepG2.2.15 cells overexpressing DYRK4, highlighting significant targets. (I) qRT-PCR analysis estimated the relative DYRK4 and FOS levels in pHBV1.3-transfected Huh7 cells with either overexpression or knockdown of DYRK4. GAPDH served as the loading control. (J) Following co-transfection of DYRK4-FLAG or shDYRK4 with the pHBV1.3 plasmids in Huh7 cells, Western blot analysis was performed to determine FOS expression levels. (K) After co-transfection of shDYRK4, and siSTAT3 in Huh7 cells, the protein levels of DYRK4, STAT3, and FOS were assessed by Western blot. (L) In Huh7 cells, Western blot analysis detected the protein levels of FOS and STAT3 upon overexpression of either wild-type DYRK4 or K133R mutant along with the pHBV1.3 plasmids. ** *P* < 0.01, *** *P* < 0.001.

To further investigate the downstream anti-HBV mechanism of DYRK4, we conducted RNA sequencing analysis with and without overexpression of DYRK4 in HepG2.2.15 cells. Our analysis revealed enrichment of the JAK-STAT signaling pathway **(Fig. 6A)**, concomitant with our mass spectrometry results of DYRK4 that found STAT3 as a potential target. Subsequently, co-IP experiment confirmed the interaction between DYRK4 and STAT3 **(Fig. 6B)**. We also observed that overexpression of DYRK4 led to a decrease in STAT3 protein levels. However, knockdown of DYRK4 had the opposite effect **(Fig. 6C)**.

Another DYRK family member, DYRK2, was previously reported to phosphorylate TBK1 at Ser527 to trigger its ubiquitination 9. The kinase activity of DYRK2 was shown to be required for the regulation of katanin degradation 24, and DYRK2 was shown to phosphorylate JUN at Ser243 or C-Myc at Ser62, which led to recruitment of an E3 ubiquitin ligase and subsequent degradation of these proteins 25. Thus, to determine if DYRK4 decreases STAT3 protein through ubiquitination-induced degradation as does DYRK2, we performed protease inhibitor experiments and demonstrated that MG132 inhibited degradation of phosphorylated STAT3 and enhanced the total STAT3 protein level under conditions of DYRK4 overexpression **(Fig. 6D)**. Further, ubiquitination up-regulation of STAT3 induced by DYRK4 was confirmed by immunoblotting **(Fig. 6E)**. However, this ubiquitination failed when K133 was mutated **(Fig. 6F)**. Subsequently, the nuclear and cytoplasmic fractionation experiments demonstrated that overexpression of DYRK4 impeded nuclear translocation of phosphorylated STAT3 **(Fig. 6G, upper panel)**, whereas DYRK4 knockdown had the opposite effect **(Fig. 6G, lower panel)**. In order to determine the molecule regulated by STAT3 during DYRK4-dependent inhibition of HBV replication, we thoroughly analyzed the RNA-sequencing data, as illustrated in the heatmap **(Fig. 6H, left panel)**. Our findings revealed that among the analyzed genes, 134 genes exhibited significant downregulation, while 140 genes showed significant upregulation **(Table S6)**. *FOS* transcription levels were substantially reduced, as indicated by the volcano plot **(Fig. 6H, right panel)**. These findings were further validated by overexpression and knockdown of DYRK4 in Huh7 cells **(Fig. 6I)** and HepG2.2.15 cells **(Fig. S6)**, which mirrored the mRNA levels of FOS observed in the RNA-sequencing data. These findings were strengthened by the DYRK4-mediated downregulation of FOS protein **(Fig. 6J)**.

Previous studies have shown that STAT3 can bind to the promoter region of *FOS*, enhancing its transcriptional activity and upregulating its expression 26. Therefore, we speculated that diminished STAT3 caused by DYRK4 also leads to decreased *FOS* transcription levels. To validate this hypothesis, we knocked down DYRK4, which resulted in an upregulation of STAT3, and an increase in FOS expression. Additionally, STAT3 knockdown resulted in decreased FOS expression **(Fig. 6K).** Since DYRK4 kinase activity is crucial in inhibiting HBV replication, we overexpressed the wild-type and K133R mutant of DYRK4 in cells and found that the wild-type DYRK4 significantly reduced both STAT3 and FOS proteins as compared to the K133R mutant **(Fig. 6L)**. These findings collectively elucidated how DYRK4 regulates HBV replication by downregulating STAT3-FOS expression.

### DYRK4 suppresses HBV replication by inhibiting autophagy through the STAT3-FOS signaling axis

**Figure 7:**
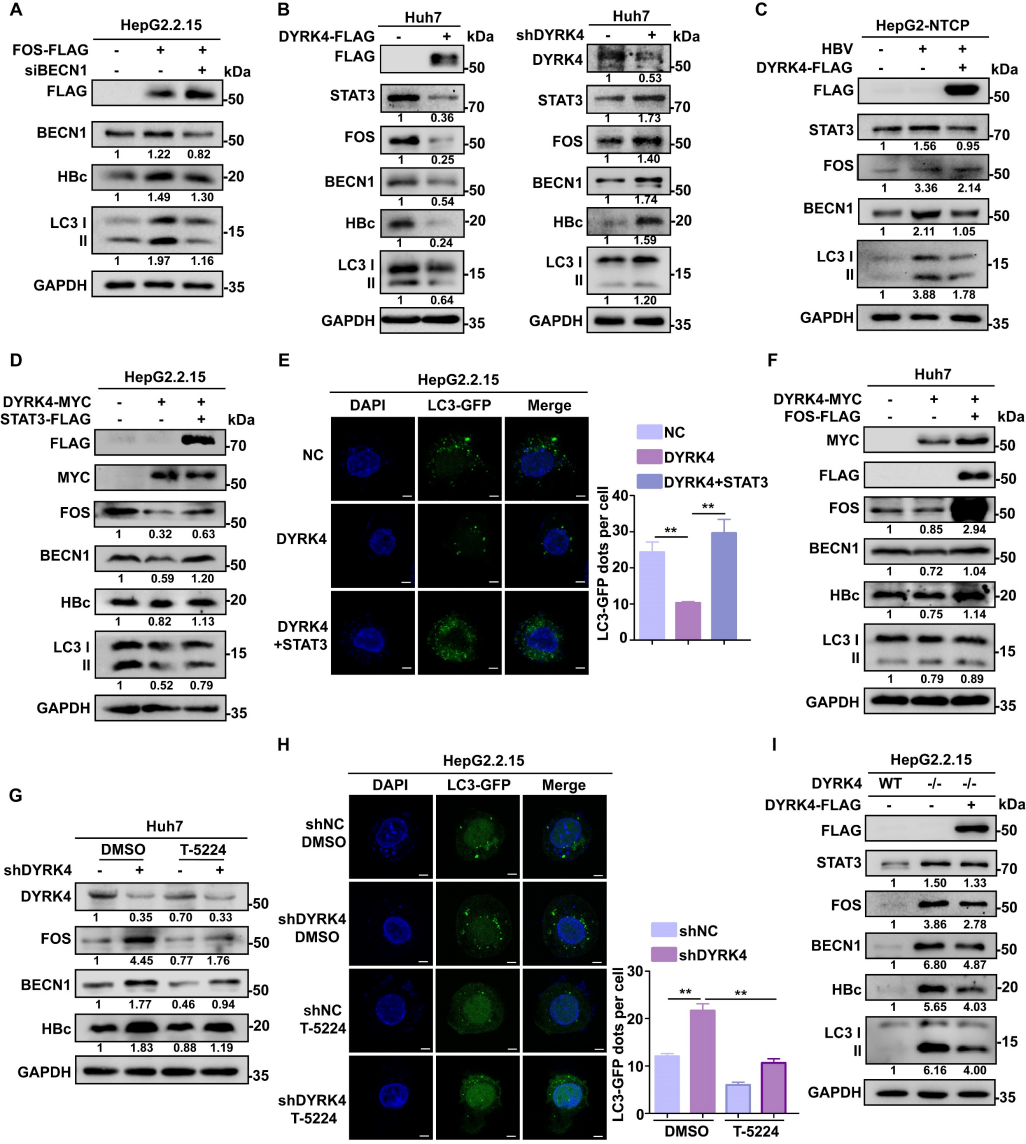
** DYRK4 suppresses HBV replication by inhibiting autophagy through the STAT3-FOS signaling axis.** (A) HepG2.2.15 cells were transfected with the FOS-FLAG plasmid, followed by siRNA to knockdown BECN1. After incubating for 48 h, the cells were subjected to Western blot analysis, in order to detect the expression of FOS, BECN1, HBc, and LC3 I/II proteins. (B) Huh7 cells were subjected to overexpression and knockdown of DYRK4 with the pHBV1.3 plasmid for 48 h, followed by detection of FOS, BECN1, HBc, and LC3 I/II protein levels using Western blot analysis. (C) HepG2-NTCP cells were infected with HBV (MOI = 200) for five days and transfected with the DYRK4-FLAG plasmid for two days. The cells were lysed for Western blot. FOS, BECN1, HBc, and LC3 I/II protein levels were detected. (D) HepG2.2.15 cells were co-transfected DYRK4-MYC with STAT3-FLAG plasmids for 48 h. Cells were collected and proteins were extracted for Western blot analysis. (E) HepG2.2.15 cells were transfected with the DYRK4-MYC plasmid and STAT3-FLAG plasmid along with the LC3-GFP plasmid for 48 h. Then the cells were observed, and the LC3-GFP dots were quantified using confocal microscopy, scale bar: 5 μm. (F) Overexpression of DYRK4 and FOS were performed in Huh7 cells to detect protein levels of FOS, BECN1, and HBc through Western blot analysis. (G) DYRK4 was knocked down in Huh7 cells transfected with pHBV1.3 for 48 h, followed by treatment with T-5224 (50 μM) for 12 h. This resulted in changes in the expression of FOS, BECN1 and HBc proteins as detected by Western blot analysis. (H) Combined knockdown of DYRK4 and T-5224 treatment in HepG2.2.15 cells led to the observation and quantification of LC3-GFP dots using confocal microscopy, scale bar: 4 μm. The number of LC3-GFP dots in three replicates was analyzed by ImageJ software. (I) CRISPR-Case9 technology was used to knock out *DYRK4* in HepG2.2.15 cells. The DYRK4-FLAG plasmid was then transfected into the *DYRK4*^-/-^ cells for 48 h. The wild-type cell line was created using negative control lentivirus treatment. Western blot analysis revealed changes in the STAT3-FOS-autophagy-HBc protein levels. ** *P* < 0.01.

Several studies have demonstrated that FOS can induce autophagy. Specifically, *FOS* can bind to the promoter of BECN1 to upregulate its transcription, thereby promoting autophagy 27. BECN1 is a core player in autophagy and constitutes a molecular platform that regulates autophagosome formation and maturation 28. The autophagy protein LC3 and HBc protein can be reduced by downregulation of BECN1 **(Fig. 7A)**.

To investigate if the inhibitory effect of DYRK4 on HBV replication depends on FOS-mediated autophagy, we co-transfected pHBV1.3 and DYRK4 plasmids in Huh7 cells. Western blot analysis revealed that overexpression of DYRK4 downregulated both STAT3 and FOS levels, thus decreasing autophagy-inducible factor BECN1 and total LC3 I/II expression, consequently reducing the expression of HBc **(Fig. 7B, left panel)**. Conversely, DYRK4 knockdown yielded opposite effects **(Fig. 7B, right panel)**. Consistent results were obtained in HBV-infected HepG2-NTCP cells. We also verified that HBV infection upregulates STAT3 expression as previously reported 29, and can promote autophagy *via* STAT3-FOS, whereas STAT3-FOS-BECN1 was significantly downregulated after DYRK4 overexpression **(Fig. 7C)**.

We then overexpressed both STAT3 and DYRK4 plasmids in HepG2.2.15 cells. After overexpression of STAT3, we found that the DYRK4-dependent inhibition of HBV was lost. STAT3 restored the DYRK4-dependent inhibition of HBV replication *via* FOS-autophagy, and HBc was rescued** (Fig. 7D)**. Moreover, the observation of confocal microscopy showed that STAT3 increased the number of autophagosomes, even though DYRK4 was overexpressed** (Fig. 7E)**. Next, to further investigate the potential relationship between DYRK4-mediated inhibition of HBV replication and FOS-BECN1-regulated autophagy, we overexpressed both FOS and DYRK4 plasmids, which restored the DYRK4-induced inhibitory effect on BECN1, LC3 I/II and HBc expression **(Fig. 7F)**. Moreover, we employed T-5224 (a specific FOS inhibitor) in DYKR4 knockdown cells. The results demonstrated that T-5224 effectively suppressed the shDYRK4-induced upregulation of FOS, BECN1, and HBc **(Fig. 7G)**. Furthermore, T-5224 treatment also inhibited the enhanced secretion of HBs and HBe **(Fig. S7)**. Confocal microscopy analysis revealed that the knockdown of DYRK4 in the DMSO group led to an increase in LC3-GFP-labeled autophagosomes. However, the addition of the T-5224 inhibitor generally reduced the fluorescence intensity associated with LC3-GFP-labeled autophagosomes **(Fig. 7H)**. Additionally, HBsAg and HBeAg secretion of HBV were also significantly inhibited by knockdown of FOS in the K133 kinase mutant **(Fig. S8)**.

To examine the important role of DYRK4 in inhibiting HBV replication, we utilized CRISPR-Cas9 technology to create the *DYRK4*^-/-^ HepG2.2.15 cell line **(Fig. S9)**. When *DYRK4* was knocked out, the expression of STAT3-FOS and autophagy proteins BECN1 and LC3I/II were significantly higher than that in wild-type HepG2.2.15 cells, as was the same for HBc antigenic proteins. In contrast, overexpression of *DYRK4* in* DYRK4*^-/-^ cells resulted in another down-regulation of these above proteins **(Fig. 7I)**.

These findings collectively suggest that DYRK4 inhibits HBV replication *via* its kinase activity by regulating STAT3-FOS-BECN1 expression levels.

### DYRK4 inhibits HBV replication* in vivo*

**Figure 8:**
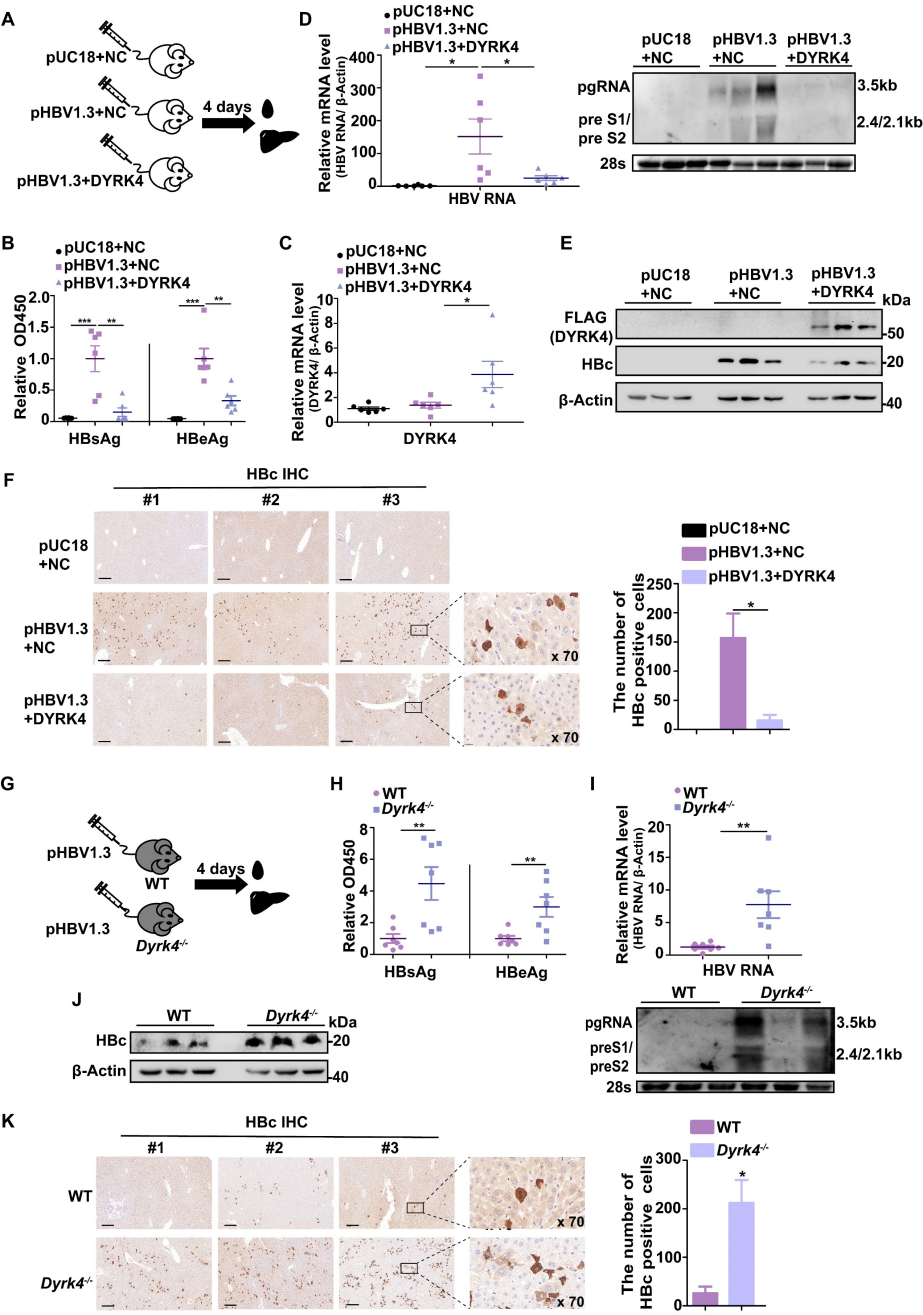
** DYRK4 inhibits HBV replication *in vivo.*** (A) The pHBV1.3 and DYRK4-FLAG plasmids (10 μg each) were hydrodynamically transfected into BALB/c mice with high pressure *via* the tail vein. Blood and liver tissue samples were collected after four days. (B) The serum HBsAg and HBeAg levels were measured by ELISA at OD450. (C) RNA was extracted from liver tissue to detect the expression of the DYRK4-FLAG plasmid by qRT-PCR. Mouse β-Actin served as the loading control. (D) Liver tissue was extracted for detection of HBV RNAs using qRT-PCR and Northern blot analysis. Mouse β-Actin served as the loading control. (E) Western blot analysis detected expression of DYRK4-FLAG and HBc proteins in the murine liver. (F) IHC staining was conducted to visualize HBc in the murine liver tissue. (G) *Dyrk4*^-/-^ C57BL/6 mice were hydrodynamically transfected with the pHBV1.3 plasmid (10 μg), followed by blood and liver tissue collection after four days. (H) ELISA was employed to assess the serum HBsAg and HBeAg levels at OD450. (I) qRT-PCR and Northern blot were performed to estimate the HBV RNAs level in the murine samples. (J) Western blot analysis revealed the expression of HBc protein in the murine liver tissues. (K) IHC staining detected the presence of HBc protein in the murine liver tissues. HBc staining was analyzed using CaseViewer and ImageJ software. The distribution of HBc in hepatocytes was observed after 70-fold magnification. Significance was indicated as follows: * *P* < 0.05, ** *P* < 0.01, and *** *P* < 0.001; scale bar: 200 μm.

Based on our observation of the association between the higher expression of DYRK4 and the inhibition of HBV replication *in vitro*, we set out to confirm the inhibitory effects of DYRK4 on HBV replication *in vivo*. Thus, we hydrodynamically injected the pHBV1.3 and DYRK4-FLAG plasmids into BALB/c mice *via* their tail vein **(Fig. 8A)**. Following four days of infection, serum analysis demonstrated that overexpressing DYRK4 reduced HBsAg and HBeAg levels **(Fig. 8B)**. Furthermore, qRT-PCR **(Fig. 8C-8D, left panel)** and Northern blot analysis **(Fig. 8D, right panel)** validated that DYRK4 downregulated HBV RNA level. Western blot and immunohistochemistry (IHC) revealed that DYRK4 decreased HBc protein levels in murine liver tissue samples** (Fig. 8E-8F)**.

Next, we generated *Dyrk4-*knockout C57BL/6 mice by targeting and splicing exons 3 to 5 of *Dyrk4* as previously described 11 **(Fig. S10A)**. Using exons specific primers, we successfully verified *Dyrk4-*knockout in mice by PCR **(Fig. S10B-S10C)** and Western blot **(Fig. S10D)**. The pHBV1.3 plasmids were then hydrodynamically injected into the WT and *Dyrk4*^-/-^ mice through their tail vein **(Fig. 8G)**. After four days, serum and liver tissue analyses of* Dyrk4*^-/-^ mice showed increased secreted viral proteins **(Fig. 8H)**, as well as transcription** (Fig. 8I, upper and lower panel).** In addition, Western blot and IHC confirmed an increase in HBc protein expression upon *Dyrk4* depletion **(Fig. 8J-8K)**. HBV replication was higher in the *Dyrk4-*knockout mice than in wild mice. These *in vivo* results demonstrated that DYRK4 effectively suppresses HBV replication similarly in two different background types of mice.

## Discussion

HBV infection remains a significant public health challenge, and patients with chronic hepatitis B face an increased lifetime risk of developing cirrhosis or HCC 30. Currently, persistent HBV infection accounts for at least 50-55% of HCC cases 31. The DYRK family, as a distinct kinase family, has been extensively associated with cellular development, innate immunity, viral infection, and liver cancer. Among the members of the DYRK family, DYRK4 has been reported to be involved in reproductive development 21, 32, cell differentiation 20, cancer occurrence 33, DNA damage regulation 34 and anti-cancer drugs 35. However, limited research exists on its role in viral infections. Importantly, our previous study showed that DYRK4 can induce antiviral innate immunity 11. Thus, in this study, we confirmed the inhibitory effect of DYRK4 on HBV replication both *in vitro* and *in vivo*. Our investigation further revealed a regulatory interaction between DYRK4 and STAT3-FOS-induced autophagy, that modulated HBV replication. Moreover, we discovered that TAB1 directly interacts with DYRK4 to enhance its autophosphorylation, which may facilitate inhibit HBV replication. Notably, we show here that the K133 kinase activity site of DYRK4 plays a crucial role in inhibiting HBV replication.

Prior studies have suggested that TAB1/2/3 typically function by forming complexes with TAK1, which activates classical pathways such as NF-κB, MAPK-JUN, and AP-1 36. However, our results showed that DYRK4 interacts with TAB1 directly, and our experiments revealed that overexpression of DYRK4 still inhibits HBV replication in *TAK1*-knockout cells **(Fig. S3)**. Under conditions of simultaneous DYRK4 and TAB1 knockdown, the inhibitory function of DYRK4 on HBV replication was significantly blocked **(Fig. 5G-5H)**. These results suggested that DYRK4 inhibits HBV replication may not rely solely on the classical TAK1-regulated pathway and may function through other signaling pathways modulated after interacting with TAB1. Further published studies have revealed that TAB1 can directly interact with p38α (MAPK14) to mediate its autophosphorylation and modulate downstream pathways 23, thereby bypassing the classical TAK1-MAPKK cascade (such as MKK3 and MKK6). TAB1 interacts with DYRK4, and may achieve autophosphorylation, enhancing its kinase activity when inhibiting HBV replication. We demonstrated that the K133 site in the DYRK4 kinase domain is crucial in this process.

As an initiating kinase, DYRK2 phosphorylates JUN Ser243 or C-Myc Ser62, which then leads to GSK3β phosphorylation of JUN Thr239 and C-Myc Thr58. This double phosphorylation enables E3 ubiquitin ligases to recognize and target JUN or C-Myc for degradation 25. Moreover, phosphorylation of TBK1 at Ser527 by DYRK2 also triggers the ubiquitin-dependent degradation of TBK1 9. The kinase activity of DYRK2 is also crucial for phosphorylating and degrading its substrate, katanin p60, thereby regulating katanin degradation through the E3 ubiquitin ligase complex 24. Our findings suggest that the kinase activity of DYRK4 may inhibit HBV replication by mediating the phosphorylation status of STAT3, which then undergoes ubiquitination-mediated degradation **(Fig. 6D-6F)**.

HBV exploits and modifies autophagy to promote the production of virions and subviral particles 37, with results in persistent autophagy activation in hepatocytes during chronic infection likely playing a key role in HBV pathogenesis 38. Some elements participating in autophagy support assembly of mature HBV core particles and subsequent envelopment of HBV particles 39. Both increased autophagosomes and blocked autophagic degradation enhance HBV replication and secretion by preventing HBV DNA degradation, thus elevating HBV offspring DNA levels 40, 41. Autophagosomes, may like endosomes, serve as alternative vesicles for HBsAg secretion and assembly 41. Additionally, the autophagy pathway promotes HBV envelopment by sequestering restriction factors and is induced by the S protein 42. Overall, this process significantly enhances HBV encapsulation, secretion, and replication 13, 43.

The *FOS* gene has been suggested to induce and activate autophagy as immediate early genes (IEGs) 44, thereby inducing autophagy by upregulating the key autophagy protein BECN1. Earlier studies have suggested that STAT3 can interact with FOS and regulate the transcription and expression of FOS by binding to the *FOS* promoter 26. Our study discovered a significant reduction in the number of autophagosomes in the presence of DYRK4 **(Fig. 2B, 2D)**. This effect may be attributed to the DYRK4-mediated downregulation of FOS expression *via* the STAT3 signaling pathway, which reduces BECN1 expression level. We further assessed how DYRK4 binding restrains STAT3 **(Fig. 6E-6G)** to inhibit the autophagic process induced by downregulating the STAT3-FOS-BECN1 axis and subsequently affecting HBV replication **(Fig. 7A-7C)**. However, further research is needed to understand the downregulation of STAT3 by DYRK4 and its impact on phosphorylation into the nucleus. A previous study showed that STAT3 can directly bind to the cccDNA of HBV 45, as well as to the HBx promoter and HBV enhancer 1 (ENh1) to promote HBV replication 46. DYRK4 promotes the degradation of STAT3, which may also contribute to HBV inhibition.

In conclusion, our results demonstrated a novel function of DYRK4 against HBV replication and gene expression. Future research on the promising therapeutic role of DYRK4 against HBV infection would be valuable.

## Supplementary Material

Supplementary method, figures and tables.

## Abbreviations

DYRK: dual-specificity tyrosine-regulated kinase
HBV: hepatitis B virus
cccDNA: covalently closed circular DNA
pgRNA: pre-genome RNA
HCC: hepatocellular carcinoma
MAP1LC3 (LC3): microtubule-associated protein 1 light chain 3
GAPDH: glyceraldehyde-3-phosphate dehydrogenase
TABs: TGF-beta activated kinase 1/MAP3K7 binding proteins
TAK1: TGF-beta activated kinase 1
STAT3: signal transducers and activators of transcription 3
