# Supplementary material for: Dual-specificity tyrosine-regulated kinase 4 modulates the STAT3-FOS signaling axis to inhibit hepatitis B virus replication via autophagy
Source: Int J Biol Sci. 2025 Mar 10;21(6):2415–29. doi: 10.7150/ijbs.105447 (PMC12035898; doi:10.7150/ijbs.105447)
Supplement: Supplementary file 1 — Supplementary method, figures and tables. [file ijbsv21p2415s1.pdf]

Supplementary materials, figures, tables to

Dual-specificity tyrosine-regulated kinase 4 modulates the STAT3-FOS signaling axis to inhibit hepatitis B virus replication *via* autophagy.

Jiaqi Xu[1], Xianhuang Zeng[1], Junsong Huang[2], Shuangshuang Ma[2], Kun Li[1], Siqi Yang[1], Wajeeha Naz[1], Tanzeel Yousaf[1], Sen Yuan[1], Yang Liu[1], Jing Zhang[1], Chaozhi Liu[2], Chenyi Liu[1], Zixu Zhai[2], Mingxiong Guo[2,3], Guihong Sun[1,4,*]

Chemicals, antibodies, and other reagents

Protease inhibitor cocktail (C0001), phosphatase inhibitor cocktail (C0004), MG132 (T2154), and T-5224 (530141-72-1) were purchased from TargetMol (China). 3-MA (HY-19312) was purchased from MCE. Antibodies to DYRK4, DYRK4 (phos-Tyr264) were purchased from Thermo Fisher. Anti-phospho-tyrosine antibody was purchased from CST. Antibodies against GAPDH, Flag-Tag (20543-1-AP), Myc-Tag (16286-1-AP), TAB1 (27566-1-AP), TAB2 (14410-1-AP), TAK1 (12330-2-AP), LC3B (14600-1-AP), BECN1 (11306-1-AP) and HRP-conjugated affinipure goat anti-rabbit IgG(H+L) (SA00001-2) were purchased from ProteinTech Group (China). Antibodies to TAB3 (A18681) and FOS (A17351) were purchased from Abclonal Technology (China). β-Actin (A5441) was purchased from Sigma. And Flag-beads (M8823) were purchased from Sigma Aldrich. An enhanced ECL chemiluminescent substrate kit was purchased from Yeasen (China). The nuclear-cytosol extraction kit P1200 was purchased from Applygen (China). The pHBV1.3 plasmid is a 1.3-fold type D HBV genome cloned into the pUC18 plasmid (Genbank number is V01460.1).

Supplementary figures

Figure S1:

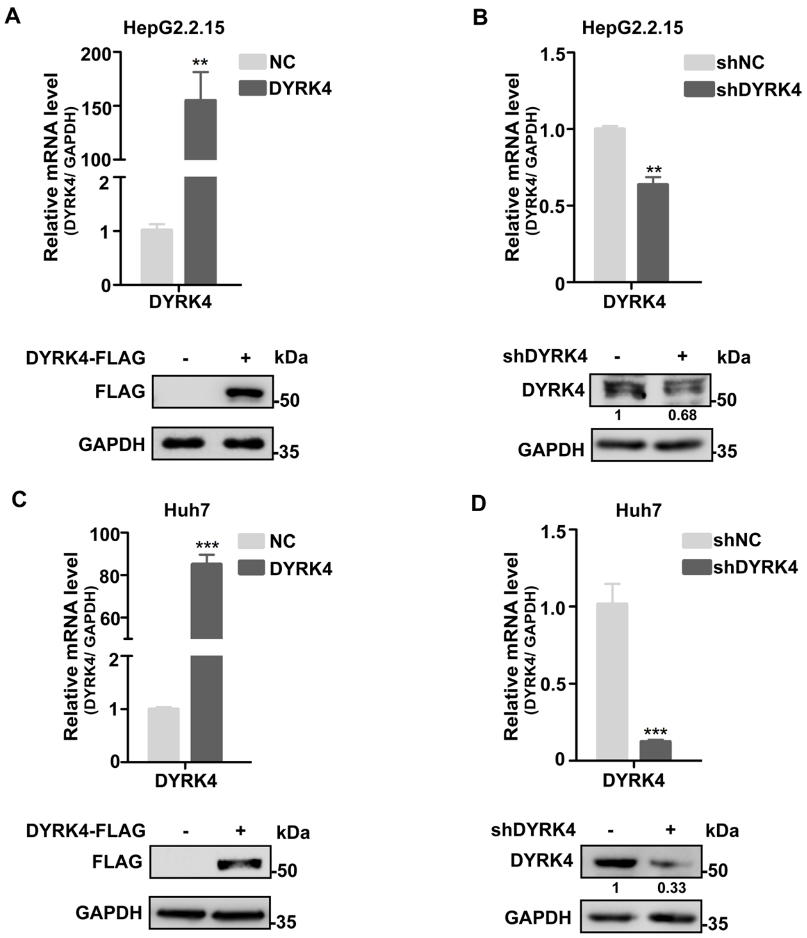

Figure S1: Detection of DYRK4 plasmids and shRNA in the HepG2.2.15 and Huh7 cell lines. (A) The DYRK4-FLAG plasmid or (B) shRNA plasmid of DYRK4 was transfected into HepG2.2.15 cells for 48 h, and the expression of the plasmid was assessed by qRT-PCR and Western blot analysis.

(C) The DYRK4-FLAG plasmid or (D) shRNA plasmid of DYRK4 was transfected into Huh7 cells with the pHBV1.3 plasmid for 48 h, and the expression of the plasmid was assessed by qRT-PCR and Western blot analysis. GAPDH served as the loading control. ** $P < 0.01$, *** $P < 0.001$.

Figure S2:

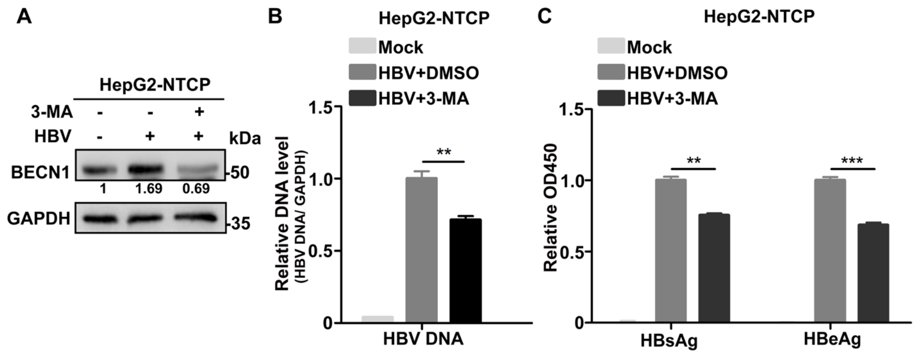

<u>Figure S2:</u> The importance of autophagy in HBV infection has been demonstrated in HepG2-NTCP cells.

HepG2-NTCP cells were infected with HBV (MOI = 200) for five days. After five days of infection, treatment with 3-MA was continued for 24 h. The cell supernatants and cells were collected. (A) BECN1 was detected by Western blot and the effect of 3-MA on autophagy was assessed. (B) The total HBV genomic DNA was extracted and quantified by qRT-PCR using a HBV DNA primer, GAPDH DNA primer served as the loading control. (C) Detection of HBsAg and HBeAg levels in cell supernatants by ELISA at OD450. ** $P < 0.01$, *** $P < 0.001$.

Figure S3:

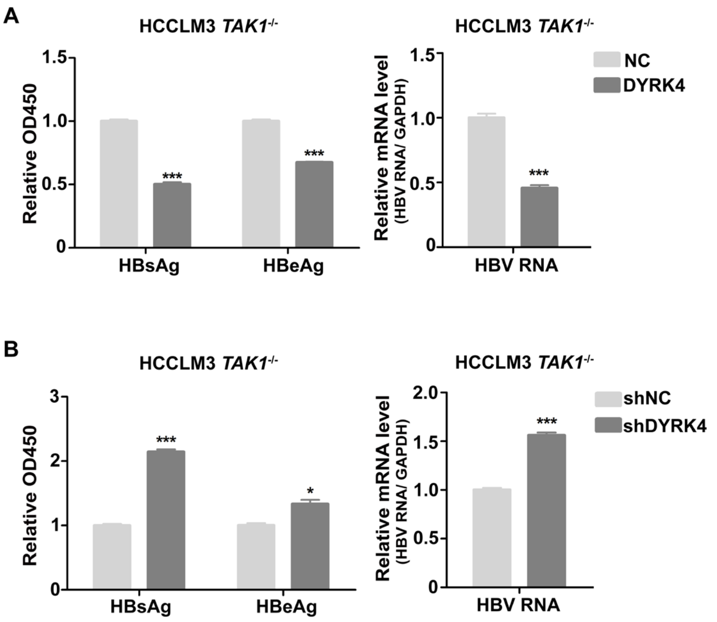

<u>Figure S3:</u> Anti-HBV function of DYRK4 in the *TAK1*$^{-/-}$ HCCLM3 cell line.

(A) The pHBV1.3 plasmid and the DYRK4-FLAG plasmid were co-transfected into the *TAK1*$^{-/-}$ HCCLM3 cell line for 48 h. RNA was extracted for qRT-PCR to detect HBV RNA, and supernatant was collected to detect HBsAg, HBeAg by ELISA.

(B) The pHBV1.3 plasmid and shDYRK4 plasmid were co-transfected into the *TAK1*$^{-/-}$ HCCLM3 cell line for 48 h. RNA was extracted for qRT-PCR to detect HBV RNA, and the supernatant was collected to detect HBsAg and HBeAg by ELISA. GAPDH served as the loading control. * $P < 0.05$, *** $P < 0.001$.

Figure S4:

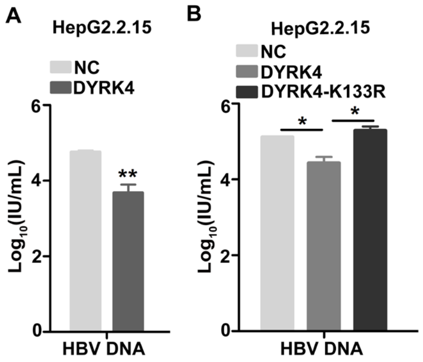

<u>Figure S4:</u> The effect of DYRK4 kinase activity on HBV DNA in the supernatant of HepG2.2.15 cells.

(A-B) The DYRK4 plasmid and the K133R plasmid of DYRK4 were transfected for 48 h. The supernatant of the HepG2.2.15 cells was collected for HBV nucleic acid extraction. Quantitative HBV DNA was detected by qRT-PCR using the HBV nucleic acid quantitative kit (Sansure Biotech, China). ** $P < 0.01$, * $P < 0.05$.

Figure S5:

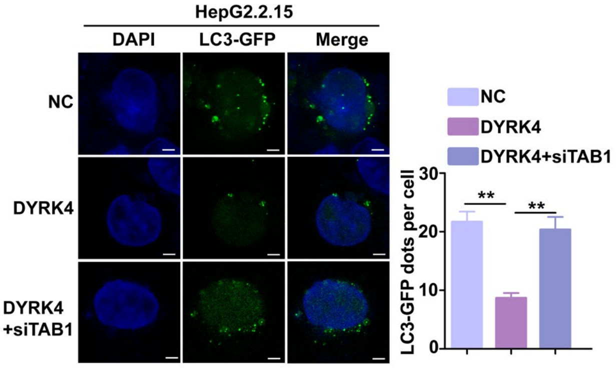

Figure S5: Knockdown of TAB1 increases autophagy.

TAB1 was knocked down after overexpression of DYRK4 by siRNA. LC3-GFP-labeled autophagosomes were observed by confocal microscopy. The LC3-GFP plasmid, DYRK4-FLAG plasmid and siTAB1were transfected together into HepG2.2.15 cells and observed 48 h later. Scale bar: 4 μm; ** $P < 0.01$.

Figure S6:

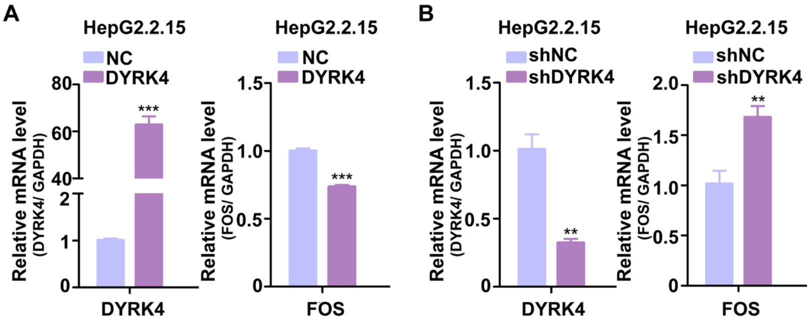

Figure S6: DYRK4 downregulates the mRNA levels of FOS in HepG2.2.15 cells.

(A) The DYRK4-FLAG plasmid was transfected into HepG2.2.15 for 48 h; the RNA was extracted for qRT-PCR.

(B) The shDYRK4 plasmid was transfected into HepG2.2.15 for 48 h; the RNA was extracted for qRT-PCR. GAPDH served as the loading control. ** $P < 0.01$, *** $P < 0.001$.

Figure S7:

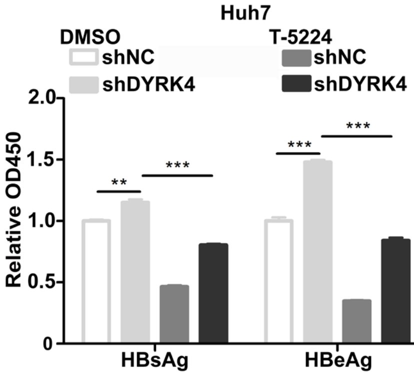

Figure S7: FOS acts as a downstream regulator of DYRK4 to regulate HBV replication. DYRK4 was knocked down in Huh7 cells transfected with pHBV1.3 for 48 h, followed by treatment with T-5224 (50 μM) for 12 h. ELISA for the detection of HBsAg and HBeAg in the supernatant after treatment with T-5224. ** *P* < 0.01, *** *P* < 0.001.

Figure S8:

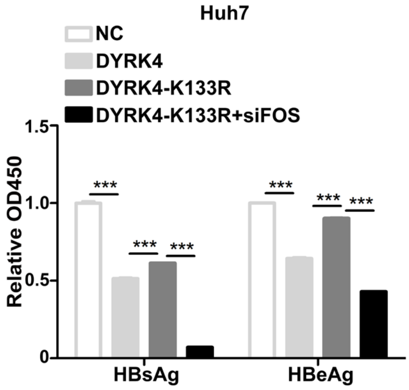

<u>Figure S8:</u> The kinase activity of DYRK4 influenced FOS to regulate HBV replication.

The overexpression experiment was performed in pHBV1.3-transfected Huh7 cells for 48 h with either wild-type DYRK4 or K133R mutant together with siFOS to examine changes in HBsAg and HBeAg by ELISA. *** $P < 0.001$.

Figure S9:

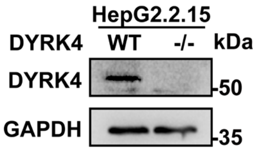

<u>Figure S9:</u> Detection of DYRK4 in WT and *DYRK4*$^{-/-}$ HepG2.2.15 cell line by Western blot.

Figure S10:

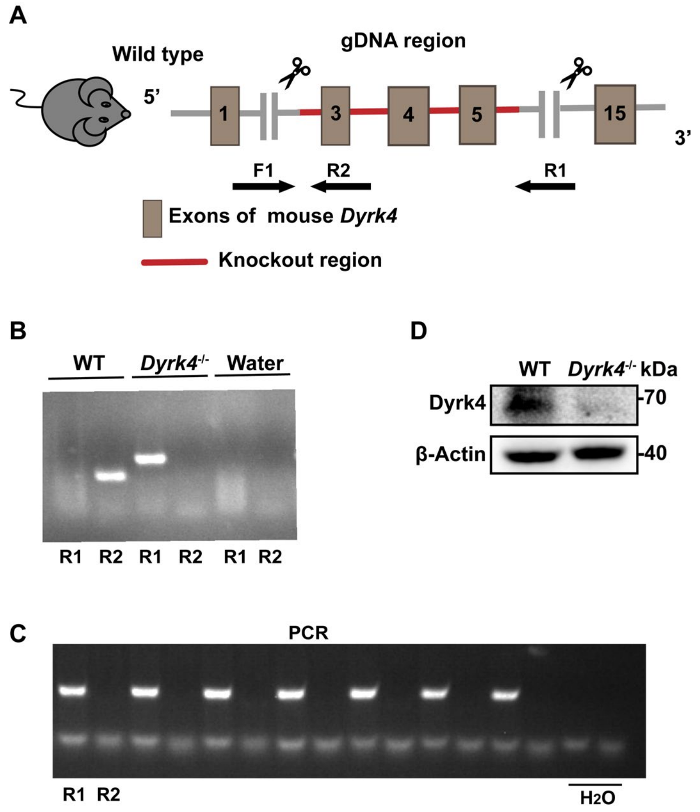

Figure S10: Transgenic C57BL/6 mice of *Dyrk4*$^{-/-}$.

(A) The *Dyrk4* gDNA region, exons 3 to 5 of C57BL/6 mice were knocked out. The upstream primer F1, downstream primer R1, and downstream primer R2 were designed. PCR was performed using both F1+R1 and F1+R2 primers simultaneously to detect whether it was a knockout mouse.

(B) Agarose gel electrophoresis. *Dyrk4*-WT mouse exhibits only the R2 band. *Dyrk4*-Knockout mouse exhibits only the R1 band. Homozygotes: 660 bp (R1); Wild type: 433 bp (R2).

(C) PCR detection of *Dyrk4*$^{-/-}$ mice before hydrodynamic injection of the pHBV1.3 plasmid.

(D) Western blot was performed to detect the Dyrk4 expression in murine liver tissue.

Supplementary Tables

Table S1 qRT-PCR primers list

Gene name	Forward primer	Reverse primer
GAPDH	5’-AGAAGGCTGGGGCTCATTTG-3’	5’-AGAAGGCTGGGGCTCATTTG-3
GAPDH (for DNA）	5’-TCGTATTGGGCGCCTGGTC-3’	5’-CGTGAGGGTATGAAGGGGC-3’
DYRK4	5’-GCTGTCATCACTCGAGCAGA-3’	5’-CTTGGGAGCGTCTACCAGTT-3’
Mus Dyrk4	5’-GCAACAAAGTCCCATCAAAGG-3’	5’-GTCTTGGGCTTTGGTGTTAATG-3’
Mus β-Actin	5’-AAGTGTGACGTTGACATCCGTAAA-3’	5’-CAGCTCAGTAACAGTCCGCCTAGA-3’
HBV RNA	5’-GCACTTCGCTTCACCTCTGC 3’	5’-CTCAAGGTCGGTCGTTGACA-3’
HBV DNA	5’-CTCGTGGTGGACTTCTCTC-3’	5’-CTGCAGGATGAAGAGGAA-3’
HBV cccDNA	5’-GCCTATTGATTGGAAAGTATGT-3’	5’-AGCTGAGGCGGTATCTA-3’
FOS	5’-CACTCCAAGCGGAGACAGAC-3’	5’-AGGTCATCAGGGATCTTGCAG-3’

Table S2 sgRNA sequences table

sgRNA	Sense	Antisense
sgDYRK4#1	5’-CACCGGATCCTGGGTTTTAATGCT-3’	5’-AAACAGCATTAAAACCCAGGATCC-3’
sgDYRK4#2	5’-CACCGCATTAAAACCCAGGATCCCA-3’	5’-AAACTGGGATCCTGGGTTTTAATGC-3’
sgTAK1	5’-CACCGCGACTACAAGGAGATCGAGG-3’	5’-AAACCCTCGATCTCCTTGTAGTCGC-3’

Table S3: siRNA sequences table

siRNA	Sense	Antisense
siNC	5’-UUCUCCGAACGUGUCACGU-3’	5’-ACGUGACACGUUCGGAGAA-3’
siTAB1#1	5’-GGAGUGAGAACAACUGCUU-3’	5’-AAGCAGUUGUUCUCACUCC-3
siTAB1#2	5’-GGAUGAGCUCUUCCGUCUU-3’	5’-AAGACGGAAGAGCUCAUCC-3’
siFOS#1	5’-GGCGUUGUGAAGACCAUGA-3’	5’-UCAUGGUCUUCACAACGCC-3’
siFOS#2	5’-CCUAUCUGGGUCCUUCUAU-3’	5’-AUAGAAGGACCCAGAUAGG-3’
siSTAT3#1	5’-GCACAAUCUACGAAGAAUCAA-3’	5’-UUGAUUCUUCGUAGAUUGUGC-3’
siSTAT3#2	5’-GCUGACCAACAAUCCCAAGAA-3’	5’-UUCUUGGGAUUGUUGGUCAGC-3’
siBECN1#1	5’-CUGGACGAGUUUCAAGA-3’	5’-CUGGACACGAGUUUCAAGA-3’
siBECN1#2	5’-GGAGUCUCUGACAGACAAA-3’	5’-UUUGUCUGUCAGAGACUCC-3’

Table S4 shRNA plasmid primer table

Gene name	Forward primer	Reverse primer
shDYRK4#1	5'-CCGGACTGGTAGACGCTCCCAAGAACTCGAGTTCTTGGGAGCGTCTACCAGTTTTTTG-3'	5'-AATTCAAAAAACTGGTAGACGCTCCCAAGAACTCGAGTTCTTGGGAGCGTCTACCAGT-3'
shDYRK4#2	5'-CCGG CCAGAAAGTATACACGTACATCTCGAATGTACGTGTATACTTTCTGGTTTTTG-3'	5'-AATTCAAAAACCAGAAAGTATACACGTACATCTCGAGATGTACGTGTATACTTTCTGG-3'
shNC	5'-CCGGTTCTCCGAACGTGTCACGTCTCGAGACGTGACACGTTCGGAGAATTTTTG-3'	5'-AATTCAAAAATTCTCCGAACGTGTCACGTCTCGAGACGTGACACGTTCGGAGAAC-3'

Table S5 Mus PCR primer table

Gene name	Forward primer	Reverse primer
Mus Dyrk4	***F1*** 5'-CTATTGAAACAGTCCCCATCAGTAC-3'	***R1*** 5'-CAGCCTCCTTCCCTAAAGCCATC-3'<br>***R2*** 5'-CGCCCAGAACTTTACCATCAATG-3

Table S6 Table of upregulated and down-regulated genes in DYRK4-overexpressed HepG2.2.15 cells (RNA-Sequencing)

**Up-regulated genes**	**Up-regulated genes**	**Up-regulated genes**
RP4−724E16.2	RP11−545M17.1	MYBL1
CTD−2002J20.1	RP11−481J2.3	C5orf51
RP11−195E2.1	FOXO3B	TSC22D3
RP11−435O5.5	ALG10B	MORC3
METTL21B	RP11−380N8.7	TAB3−AS2
SYDE2	AF127936.9	RP11−57H14.2
OTUD1	RNF32	RP11−53I6.3
RP11−406H23.2	CTD−2024P10.1	SPG20−AS1
RP4−742J24.2	FAM171B	PRSS23
CTA−246H3.11	SP4	HAVCR1
RP11−429P3.3	RP11−114H24.7	RP1−228H13.5
HSPA6	AC008592.3	APH1B
AC007899.3	SLC45A1	RP11−298J20.4
SMG1P7	DYRK4	BAALC−AS1
RP11−513D5.5	RP11−500M8.7	SETD9
PPP1R26−AS1	CTH	CTC−459F4.1
AC019097.7	CTD−2154I11.2	TCAF1P1
E2F7	TUG1_3	TBL1Y
TTC21B−AS1	CTB−13L3.1	RP11−188P20.3

RP11−515O17.2	RP11−69I8.3	RP11−729L2.2
SLC39A10	TRIM2	ZBTB33
RB1	RP11−98I9.4	CD2AP
NRIP1	SIRT1	AC079305.10
ARL13B	ZNF708	DDX21
AC078883.4	CCR10	TMPO−AS1
CH17−232I21.1	HIPK3	ZNF25
LINC00936	DSG2−AS1	FAM13B
LIN28B	NEK7	RP11−77G23.2
AC010226.4	TNKS2	CTD−2031P19.5
CTA−797E19.2	HIF1A−AS1	KAT6B
RAPGEF6	PRKAR2A−AS1	RAD21−AS1
CTC−432M15.3	TRUB1	PKD2L2
RP11−961A15.3	RP11−485O10.3	RP11−507K12.1
ADGRV1	ZDBF2	RP11−211G3.2
AC007098.1	IL6ST	ELL2P1
XXbac−BPG181M17.6	NCOA7	RP11−1035H13.2
LINS1	NCOA2	ZNF674
RP4−621N11.2	RP11−259K5.2	NXT2
HSP90AB4P	FOXN2	AC007271.3
NOS1AP	RP11−378A13.2	ARNTL
NCR3LG1	ZNF280B	BMS1P2
SPIN4	AC078883.3	RP11−138I18.1
RP11−119K6.6	ZBED6	AC099850.1
RP11−181C21.4	AC008063.2	CTD−2368P22.1
SLC7A11−AS1	EEF1A1P19	AC017101.10
ATP7A	AC083949.1	STX18−AS1
RP4−694A7.2	AC005540.3	

**Down-regulated genes**	**Down -regulated genes**	**Down -regulated genes**
PQLC3	DSCAML1	CDH12P2
WNT11	PLA2G4C	BATF
EFCAB10	HIST1H2AC	AXL
RP11−806L2.2	DKFZP434K028	B9D2
SOCS2	AC005532.5	CTAGE3P
NLGN3	PHLDA2	ARRDC3
DKK1	ALMS1−IT1	RP11−84A14.5
TRAC	RP11−115D19.1	FGF17
OR2I1P	RP13−258O15.1	CTD−2184D3.5
SELPLG	RP11−54O7.17	RP11−216B9.6
KRT19	RNA5−8S5	RRAD
A4GALT	VTRNA1−2	AREG
HK1	PHYHIP	S100A4

NUAK1	LLNLR−304A6.2	LOXL4
ANKRD24	SNORD54	RP11−334J6.6
LCN12	LDLRAD2	PECAM1
S100A11	PFKP	LINC00973
ABRACL	ARHGDIB	ALX3
RP11−49O14.2	SCARNA17	RP11−401P9.5
TOPORS−AS1	FSTL3	RP11−368P15.3
FASLG	RP11−638I2.6	FER1L4
AC004012.1	KRT7	PLK2
RP11−165J3.6	IFITM10	AMPD3
IL11	CSPG4	AQP3
ALS2CR12	CALHM1	RP11−543P15.1
DUSP8	AC006547.13	PCOLCE
CTD−3184A7.4	GBP4	ZDHHC11B
SH2D5	DNAH1	NYAP1
C10orf91	RPL5P23	GIPR
TNNT2	IKZF3	ODF3B
RP11−542C16.1	LINC00482	C17orf107
FOXH1	GIMAP4	RP11−166P13.3
RPL17P50	RP11−727F15.12	TMEM105
NKX6−2	SERTAD1	MIR6859−2
RP11−111M22.3	RGS20	TFF1
IGFBP6	PLPP7	CCDC74BP1
PRX	FABP3	CYR61
DICER1−AS1	RP11−458F8.4	AMH
REC8	HSPE1P2	RP11−73M7.6
C2orf76	GABBR1	UBD
SLC25A27	RP11−284F21.10	RN7SL1
ITK	FOS	RP11−566K11.4
U4	LAGE3	KRT80
CGN	CHRNE	A1BG
ANO9	RP11−84C10.4	
